# Pro-Healthy Diet Properties and Its Determinants among Aging Masters Athletes

**DOI:** 10.3390/ijerph18147614

**Published:** 2021-07-17

**Authors:** Joanna Ratajczak, Urszula Czerniak, Dariusz Wieliński, Monika Ciekot-Sołtysiak, Jacek Zieliński, Piotr Gronek, Anna Demuth

**Affiliations:** 1 Department of Anthropology and Biometry, Poznan University of Physical Education, 61-871 Poznań, Poland; czerniak@awf.poznan.pl (U.C.); wielinski@awf.poznan.pl (D.W.); demuth@awf.poznan.pl (A.D.); 2 Department of Athletics, Strength and Conditioning, Poznan University of Physical Education, 61-871 Poznań, Poland; ciekot@awf.poznan.pl (M.C.-S.); jzielinski@awf.poznan.pl (J.Z.); 3 Department of Dance, Poznan University of Physical Education, 61-871 Poznań, Poland; gronek@awf.poznan.pl

**Keywords:** aging athletes, masters athletes, diet quality, diet quality indicator

## Abstract

Qualitative dietary assessments are not common in aging athletes. Therefore, this study aimed to evaluate diet quality and its determinants among aging masters athletes. Eighty-six participants of the 8th World Masters Indoor Athletics Championships were enrolled in the study (age range 36–65 years). Three subgroups were distinguished to represent countries with different eating habits. Body composition was measured by bioelectrical impedance. Eating habits and diet quality were assessed using the Dietary Habits and Nutrition Beliefs Questionnaire (KomPAN^®^, Warszawa, Poland), and the Pro-healthy Diet Index (pHDI-10). Dietary quality determinants were identified by a multiple regression model conducted for each subgroup separately (Great Britain, France, and Poland). The results showed that none of the subgroups adhered to the reference intake of products with beneficial health outcomes. This was particularly noticeable in the insufficient consumption of whole grain products, dairy, and fish. The fish and vegetables consumption frequency significantly differentiated the eating habits of the studied groups. Diet quality determinants varied depending on the group. However, in each of them, fruit consumption was one of the components of a good-quality diet. The obtained results can be used by institutions providing health education among the elderly to develop an appropriate strategy aimed at changing inappropriate eating habits.

## 1. Introduction

Rational diet is of great importance for health and achieving satisfactory sports performance [1,2,3,4], and therefore, proper nutrition should become a significant element of a healthy lifestyle. Food choices are determined by a wide range of factors, among which age, experience with food, flavor preferences, knowledge, beliefs, as well as geographical location and cultural context are mentioned most frequently [5,6]. Unfortunately, incorrect food choices are common, and non-adherence to dietary recommendations has been noted in various groups directly related to sport. Thus far, inadequate food choices were confirmed in physical education students from different countries [7,8,9,10,11,12] and in professional and semi-professional athletes [13,14,15,16].

In this regard, much less attention has been paid to masters athletes who are considered the epitome of healthy aging, as well as sports role models [17]. These are people over 35 years of age, usually experienced sportsmen and women, but also those who return to sport after a certain period of inactivity or recreational athletes [18]. All of them attempt to maintain their level of endurance as long as possible through training and participation in competitions organized specially for them [19]. Although their unusual capacity to maintain physical performance has been broadly studied [20,21,22,23,24], the knowledge about their dietary habits and diet quality is sparse.

According to the literature, the research on nutrition quality mainly included young athletes. Most of them focused on the nutrient-based approach, measuring energy and single nutrient intake and comparing them with specific recommendations or population dietary references [25,26,27,28,29,30,31,32,33,34]. However, this approach gives a poor insight into diet quality and health outcomes [35]. A better insight into diet quality and explanation of diet-health relations is provided by the food-based approach [36] in which the whole dietary patterns, i.e., the variety, frequency, and quantity with which the foods are habitually consumed, are considered [37]. According to this approach, the quality of the diet is evaluated using indicators that assess the variety of healthy choices within basic food groups and the compliance of eating patterns with nutritional guidelines [38]. There are a large number of diet quality indicators (DQIs), and some of them, i.e., the Healthy Eating Index (HEI), Athlete Diet Index (ADI), Pro-healthy Diet Index-10 (pHDI-10), and Australian Eating Surveys (AES), have been used to evaluate the quality of the diet of young athletes [12,39,40,41,42,43,44,45]. However the literature review conducted in Web of Science, PubMed, and Google Scholar databases, using the keywords “masters athletes OR masters athletics AND diet quality OR diet quality indicator OR dietary assessment” has shown that a qualitative assessment of diet and eating habits has not yet been conducted in the group of masters athletes.

Considering the importance of food choices for health and sport performance and given the lack of data specific to masters athletes, the purpose of this study was to evaluate the quality of the diet (related to the frequency of consumption of products with potentially beneficial effects), as well as its correlates in masters athletes from selected European countries.

## 2. Materials and Methods

### 2.1. Ethical Approval

The study was approved by the Bioethics Committee at Poznań University of Medical Sciences, resolution no. 203/19 (7 February 2019). All participants provided written informed consent prior to their participation in this study.

### 2.2. Sample and Study Design

The cross-sectional study was conducted in Toruń (Poland) in 2019 during the 8th World Masters Indoor Athletics Championships. Initially, the study enrolled both women and men, but the percentage of women who agreed to participate in the study was too low to allow reliable statistical analyzes. Finally, the study included 86 male masters athletics (MA, 61 from Poland, 14 from France, and 21 from Great Britain) aged 35–65 years. The choice of the research group was influenced by the number of athletes from a given country and their cultural differences in terms of eating habits. The study was conducted by trained persons. A standard measuring technique was used to perform the following measurements: height measured with an anthropometer (accuracy ±1 mm), body mass measured with a digital scale (±100 g), and body composition measured with the use of a bioelectrical impedance method. Tanita MC 980 was used to determine the percentage and kilogram values of body fat (BF), fat free mass (FFM), muscle mass (MM), and bone mass (BM). BMI was calculated and classified according to the WHO criteria.

Food consumption data were collected using the Dietary Habits and Nutrition Beliefs Questionnaire (KomPAN^®^) intended for people aged 15–65 years [46]. The study was conducted in the form of a direct individual questionnaire interview. The KomPAN^®^ questionnaire gathered information on the self-assessment of the diet, the number of meals consumed daily, and the determination of the pro-Healthy Diet Index-10 (pHDI-10), which provides information about diet quality. The index is the total of the daily consumption frequency (times/day) of the 10 food groups with potentially beneficial products: 1. whole meal bread; 2. grains and coarse-ground groats; 3. milk (including flavored milk, cocoa, coffee with milk); 4. fermented milk beverages; 5. curd; 6. white meat; 7. fish; 8. legumes; 9. fruit; 10. vegetables. Each respondent reported habitual consumption of the above-mentioned products by indicating one of the six frequency categories: never, 1–3 times a month, once a week, a few times a week, once a day, a few times a day. Those categories were converted to daily frequency expressed as times/day: never (0), 1–3 times a month (0.06), once a week (0.14), a few times a week (0.5), once a day (1.0), a few times a day (2.0). The values of the index ranged from 0–20 points, but the authors of the questionnaire recommended expressing the pHDI-10 with points on a 0–100 scale based on the formula presented below. The pHDI-10 values in the range of 0–33 points were defined as low, in the range of 34–66 points as moderate, and in the range of 67–100 points as high. The interpretation of the index is as follows: the higher the value, the greater the intensity of health-promoting properties in the diet and, therefore, the better the diet quality [47].

$$\mathrm{pHDI}-10\;\mathrm{in}\;\mathrm{points}=\frac{100}{20}\;\times \;\mathrm{sum}\;\mathrm{of}\;\mathrm{the}\;\mathrm{consumption}\;\mathrm{of}\;10\;\mathrm{food}\;\mathrm{groups}\;(\mathrm{times}/\mathrm{day})$$

### 2.3. Data Analysis

All statistical analyses were performed using the STATISTICA 13 (Dell Inc.; Tulsa, OK, USA, StatSoft Polska, Cracow, Poland, 2017). The normal distribution of the analyzed variables was verified using the Kolmogorov–Smirnov test. The threshold of statistical significance was set at *p* < 0.05. The arithmetic mean and standard deviation (SD) were calculated for sport-specific and body composition variables. Comparison analyses for continuous variables were tested with the analysis of variance (ANOVA, *F*) or its nonparametric counterpart, the Kruskal Wallis’ test (*H*). The chi-square test (χ^2^) was applied for comparative analysis of categorical variables. The median, lower, and upper quartiles were calculated for consuming the 10 product groups, pHDI-10 scores, age, and the number of daily consumed meals. The Spearman’s rank correlation coefficients (*r*) were used to assess the presence and strength of the relationship between diet quality and consumption of selected products. The interpretation of the correlation coefficients was as follows: weak (<0.3), moderate (0.3 to <0.5), strong (0.5 < 0.7), and very strong (≥0.7) correlation [48]. To identify the determinants of diet quality in each distinguished group, three separate multiple regression models were conducted with diet quality as a dependent variable. Only factors that correlated significantly with diet quality were included in the models.

## 3. Results

### 3.1. Group Characteristics

The characteristics of the distinguished groups are presented in Table 1. The study enrolled 86 men, and the mean age was approximately 50 years old. The distinguished subgroups were homogenous in terms of competition experience and body composition variables. The onset of MA career was between 41 and 43 years of age, and its average duration was in the range of 7–8 years depending on the group. The athletes’ body height ranged from 177.4 cm to 177.9 cm, and weight ranged from 75.5 kg to 77.9 kg. The mean BMI values were within the normal range. According to WHO criteria, 37% of participants were overweight or obese. The prevalence of overweight and obesity did not differ between groups (χ^2^ = 0.236, *p* = 0.888). The body fat percentage ranged from 15.2% to 16.7%. The fat free mass ranged from 63.7 kg to 65.4 kg, and muscle mass from 60.6 kg to 62.1 kg.

### 3.2. Diet Characteristics

Most of the participants (89%) described their diet as good or very good. In the nutrition of surveyed athletes, the four-meal model was dominating (43%), which was followed by a three-meal model (43%). Out of the surveyed group, no athletes consumed two or fewer meals per day. In Polish masters athletics, a significant negative correlation between the number of daily consumed meals and age was observed (r = −0.338, *p* = 0.015). The pHDI-10 value did not differ significantly between the studied groups (Poland: 25.50 points, France: 29.75 points, Great Britain: 31.00 points, F = 1.047; *p* = 0.355). In 65% of participants, the quality of the diet was low, and there were no athletes with high-quality diets. The occurrence of particular classes of diet quality (low/moderate/high) did not differ between groups (χ^2^ = 0.030, *p* = 0.985).

In each of the studied groups, fruit and vegetables were consumed most frequently, while products with the lowest consumption rates were different depending on the country (median, Q_1_ and Q_3_ in Table 2, Table 3 and Table 4). Among Polish competitors, seven products were consumed with the same lowest frequency, i.e., wholemeal bread, grains and coarse-ground groats, cottage cheese, fish, and legumes. French athletes consumed milk with the least frequency, and among British athletes, the least consumed products were milk and legumes. Significant differences were found in the consumption frequency of fish (H = 13.463; *p* = 0.001) and vegetables (H =11.476; *p* = 0.003). Additional analyses showed that Polish athletes consumed fish significantly less often than French athletes (once a week vs. a few times a week) and vegetables less often than athletes from both France and Great Britain (once a day vs. a few times a day).

### 3.3. Preliminary Analyses

Prior to testing the hypothesis regarding the correlates of diet quality among distinguished groups, we analyzed the correlations between diet quality as a dependent variable and the 10 potentially beneficial food products, as well as age and the number of daily consumed meals. In each group, moderate, albeit significant, strong and very strong correlations between diet quality and the mentioned independent variables were found. In Polish athletes (Table 2), all variables except age were positively correlated with diet quality. They were as follows: number of meals daily (r = 0.55, *p* < 0.001); wholemeal bread (r = 0.45, *p* < 0.001); grains and groats (r = 0.57, *p* < 0.001); milk (r = 0.51, *p* < 0.001); fermented milk beverages (r = 0.57, *p* < 0.001); curd (r = 0.38, *p* = 0.006); white meat (r = 0.36, *p* = 0.010); fish (r = 0.38, *p* = 0.006); legumes (r = 0.32, *p* = 0.022); fruits (r = 0.76, *p* < 0.001); and vegetables (r = 0.68, *p* < 0.001). In French athletes (Table 3), six products were positively associated with diet quality. They were as follows: wholemeal bread (r = 0.64, *p* = 0.013); fermented milk beverages (r = 0.78, *p* < 0.001); curd (r = 0.82, *p* < 0.001); white meat (r = 0.72, *p* = 0.004) legumes (r = 0.66, *p* = 0.010); and fruit (r = 0.57, *p* = 0.035). In athletes from Great Britain (Table 3), only fruit (r = 0.61, *p* = 0.003) and vegetables (r = 0.52, *p* = 0.016) were associated with diet quality. In each group, all variables that significantly correlated with the dependent variable were used to create a multiple regression model.

### 3.4. Multiple Regression Models

For each distinguished group, a regression model was conducted with diet quality as a dependent variable (Table 5). Finally, in a group of Polish masters athletics, diet quality was predicted by eight variables: fruit, grains and coarse-ground groats, curd, vegetables, milk, wholemeal bread, white meat, and fermented milk beverages. The model was significant and explained the variance of diet quality in 99.2% (*F*(8,42) = 645.67; *p* ≤ 0.001). Fruit and grains and groats consumption made the greatest contribution to the prediction of the dependent variable (respectively: ∆R^2^ = 0.546; *p* ≤ 0.001; ∆R^2^ = 0.154; *p* ≤ 0.001).

The final model of diet quality of the French masters athletes included three variables: curd, white meat, and fruit. In the first step, curd was included in the model (R^2^ = 0.736, *p* ≤ 0.001). Then, white meat was added (∆R^2^ = 0.094, *p* = 0.031) followed by fruit (∆R^2^ = 0.068, *p* = 0.027). The final model was significant and explained the variance in the dependent variable in 89.8% (*F*(3,10) = 29.31, *p* ≤ 0.001).

According to the third model, diet quality of British masters athletics was predicted by two variables: fruit and vegetables. The final model was significant and explained more than half of the variance in diet quality (R^2^ = 0.522, *F*(2,18) = 9.83, *p* = 0.001). Fruit and vegetables contributed to explaining the variance in the dependent variable in, respectively, 40.2% (*p* = 0.003) and 12% (*p* = 0.047).

## 4. Discussion

This study showed that the studied group of masters athletics is a homogeneous group in terms of competition experience and body composition variables. However, it varied in relation to certain dietary patterns. The diet quality assessment based on the consumption frequency of 10 groups of products with well-established positions in the guidelines for healthy eating [49,50] indicated numerous nutritional mistakes, which resulted in low pro-health properties of the diet. Thus far, the inadequacy of food choices has been reported in young sport-related groups [10,11,12,13,14]. However, our study was the first to show that poor food choices are also a common problem at the level of masters athletes. and that the diet quality of the studied groups is unsatisfactory. The proper level of the body components of the studied athletes may, however, indicate a well-planned nutritional strategy in terms of energy balance supported by a systematically undertaken physical activity.

A good diet is one of the foundations of good health and a condition that is especially important for athletes and their sports performance. Therefore, care for the good quality of the diet and adherence to it should be of interest to athletes at all sports levels. Unfortunately, there is no single nutritional strategy in sport [19]. Dietary recommendations for athletes vary depending on age, sport discipline, and level of physical activity, and data on the nutritional requirements of aging athletes is sparse. Despite the exceptional physical abilities of masters athletes, they undergo typical biological changes associated with the aging of the body, which in turn can modify their nutritional needs. Nevertheless, in sport nutrition, special attention should be paid to the following: balanced energy expenditure (energy intake minus energy expenditure during exercise), protein intake with every meal and its adequate supply after training, higher carbohydrate intake, the importance of vitamin D, and an individual hydration plan.

The belief in the good and very good quality of the diet among the surveyed athletes was not reflected in the results of the KomPAN^®^ questionnaire. The number of meals consumed during a day plays an important role in rational nutrition. The most advantageous for adults with different levels of physical activity is a four-meal model that ensures the maintenance of a constant blood glucose level, protecting the body against a drop in concentration, a feeling of heaviness, and fatigue [51]. In the discussed study, the four-meal model was dominant in the nutrition of athletes (44.2%), which was followed by the three-meal model (43%). These results were partially consistent with those of Czaja et al. [52] in which the dominant nutrition models in female athletics were the three- and four-meal models (37.5% each) and, in men athletics, the three-meal model (52.6%).

Food products, such as fruit, vegetables, fish, whole grains, and legumes, are considered the critical ingredients for healthy aging [53]. Unfortunately, in this study, participants did not often choose products with beneficial health outcomes, which was consistent with the results of Popławska et al. [10]. In most of the surveyed masters athletes, diet quality was low and showed weak pro-health properties, which was the result of incorrect nutritional decisions. Surprisingly, there were no athletes with a high-quality diet. Contrary to the findings of Valdes-Badilla et al. [54] and Gacek et al. [12], in the discussed research, the consumption of fruit and vegetables was the most frequent, which is significant, as in many nutritional recommendations fruit and vegetables are the basics of a healthy diet mainly due to their concentrations of vitamins, minerals, and antioxidants [55].

Unfortunately, the remaining food products were consumed with unsatisfactory frequency, and the indicated nutritional mistakes were particularly related to insufficient consumption of whole grain products, dairy, and fish. These data are largely consistent with the results of Gacek et al. [12], where students showed, among other things, insufficient consumption of whole grain cereals and fermented dairy products. Considering that whole grain products (i.e., wholemeal bread, groats, and oat flakes) are a good source of fiber and have a positive effect on the prebiotic index [56], a well-balanced diet should be rich in that type of food product. In this study, the recommendation of several portions of whole grain products daily [57] was met by less than half of the surveyed athletes (47.7%). Dairy products should also be an important supplement to the daily diet, as they are a source of wholesome protein, easily digestible calcium, and have probiotic properties [58]. The daily intake of dairy products is at least two servings of milk, which can be replaced with yogurt, kefir, or, partially, curd [59]. Our study showed that milk consumption is insufficient among studied groups of masters athletics. Suggested daily intakes were followed by only 18% of Polish athletes, 24% of athletes from Great Britain, and none of the athletes from France. Additionally, the consumption of the recommended two portions of fish per week was declared by only 18% of Polish athletes, 57% of French athletes, and 29% of British athletes.

In line with previous research [11,60], certain statistically significant differences in dietary patterns have also been revealed in this study. The frequency of consumption of fish and vegetables differentiated the eating habits in the studied groups. Polish athletes consumed fish significantly less often than French athletes, and vegetables were consumed less often than athletes from both France and Great Britain. Further evidence for the significant role of geographical factors affecting diet quality was obtained when assessing its determinants. Our research showed that the determinants differed between the countries, although in each of the studied groups, fruit consumption was one of the decisive factors for a good diet quality. This shows that an adequate supply of fruit is important in the daily diet. Among Polish masters athletics, the quality of the diet was particularly related to the consumption of eight out of the 10 products with potentially beneficial outcomes, i.e., fruit, grains and coarse-ground groats, curd, vegetables, milk, wholemeal bread, white meat, and fermented milk beverages. The regression model with all these mentioned food products explained 99.2% of the variance in the dependent variable, whereby fruit and grains and coarse-ground groats consumption accounted for 70% of this variability. In French athletes, curd, white meat, and fruit occurred as the strongest determinants of diet quality. Overall, the consumption of these products explained 89.8% of the variance in diet quality, of which almost 74% was explained by curd consumption. In British athletes, diet quality was significantly associated with fruit and vegetables, the consumption of which predicted the dependent variable in 52.2%. The diet quality was not related to age or the number of daily consumed meals in any of the studied groups. These findings stay in opposition to the data published by Schröder et al. [61] and Andrade et al. [62], which showed improving eating habits and diet quality with age.

This study has some strengths and limitations. This study was the first to qualitatively evaluate the diet of masters athletics and identify its correlates. The study was conducted in the form of a direct questionnaire interview, which allowed us to increase the understanding of the questions and obtain more complete and reliable information about the eating habits of the surveyed athletes. Whilst there are few studies assessing the quality of the diet using the pro-Healthy Diet Index-10 (pHDI-10), nevertheless, there is no doubt that eating foods included in the index has a beneficial effect on health because these are products recommended in the Mediterranean diet. The study did not consider socio-economic data, and these could influence the diet quality model in each studied group. Future research should include this type of data to gain a complete insight into the complex model of diet quality determinants. We are aware of the small size of the studied group, but this resulted from the availability of research material. We made additional analyses that showed that a change in the size of the group would not have had a significant impact on the obtained results. The current study has practical implications, as well. The obtained results can be used by institutions providing health education among the elderly to develop an appropriate strategy aimed at changing inappropriate eating habits. Education on the principles of healthy eating should be conducted and financed by people and institutions responsible for educating society from the early school years. A healthy person is a determinant of the social and economic development of the country. The improvement of knowledge in this area may be positively influenced by the wider availability of free dietary consultations, school education, increased media interest in nutrition and food, a greater number of educational programs on public television, in addition to an increase in the number of popular science publications and the promotion of reliable authorities in the field of food and nutrition. The role of educational institutions for the elderly should be fulfilled by the Universities of the Third Age, established not only in large cities but also in small and rural areas. In turn, in sport, sports clubs and associations should be responsible for a nutritional strategy supervised and implemented by specialists in the field of sports nutrition [63].

## 5. Conclusions

The study concludes that studied groups of masters athletes made improper dietary decisions, which ultimately contributed to the low diet quality in the vast majority of them. None of the studied groups adhered to the reference intake of products with beneficial health outcomes, and this was particularly noticeable in the insufficient consumption of whole grain products, dairy, and fish. Statistically significant differences in the frequency of consumption of fish and vegetables were noted. The determinants of diet quality varied depending on the group, but in each of them, the consumption of fruit was one of the components of a good-quality diet. Although this study allowed us to identify the determinants of diet quality among distinguished groups, it did not consider the socio-economic factors that could influence nutritional decisions. To better elucidate the complex model of dietary quality determinants, future research should include socio-economic data. The current study has practical implications as the obtained results can be used by institutions providing health education among the elderly to develop an appropriate strategy aimed at changing inappropriate eating habits.

## Figures and Tables

**Table 1:** Characteristics of masters athletics (arithmetic mean ± SD).

Variables	Poland *n* = 51	France *n* = 14	Great Britain *n* = 21	*p* Value
age (years)	50.5 ± 8.3	51.1 ± 9.6	50.5 ± 8.7	0.996 ^a^
onset of MA career (age)	42.1 ± 7.6	41.8 ± 7.9	43.9 ± 5.6	0.255 ^a^
duration of MA career (years)	8.4 ± 6.7	7.6 ± 5.9	7.1 ± 6.4	0.699 ^a^
height (cm)	177.4 ± 6.7	177.9 ± 4.9	177.5 ± 6.0	0.960 ^b^
body weight (kg)	77.6 ± 9.4	77.9 ± 5.8	75.5 ± 9.0	0.618 ^b^
BMI (kg/m^2^)	24.6 ± 2.4	24.6 ± 1.8	24.0 ± 2.4	0.522 ^b^
body fat (%)	16.7 ± 4.4	16.0 ± 4.2	15.2 ± 4.0	0.409 ^b^
fat free mass (kg)	64.4 ± 6.1	65.4 ± 4.4	63.7 ± 5.8	0.730 ^b^
muscle mass (kg)	61.2 ± 5.8	62.1 ± 4.2	60.6 ± 5.5	0.733 ^b^

^a^—Kruskal Wallis’ test, ^b^—ANOVA; *p* < 0.05—a statistically significant value; MA—masters athletics.

**Table 2:** Determinants of diet quality of masters athletics from Poland.

	1.	2.	3.	4.	5.	6.	7.	8.	9.	10.	11.	12.	13.
1. diet quality	-												
2. age	−0.15	-											
3. number of meals daily	0.55 *	−0.34 *	-										
4. wholemeal bread	0.45 *	−0.01	0.28 *	-									
5. grains and groats	0.57 *	−0.20	0.22	0.14	-								
6. milk	0.51 *	−0.06	0.28 *	0.16	0.19	-							
7. fermented milk beverages	0.57 *	−0.14	0.39 *	0.06	0.42	0.33 *	-						
8. curd	0.38 *	−0.05	0.21	0.12	0.08	0.36	0.20	-					
9. white meat	0.36 *	−0.17	0.27	0.07	0.07	−0.02	0.07	−0.01	-				
10. fish	0.38 *	−0.07	0.26	0.13	0.18	0.03	0.07	0.07	0.34 *	-			
11. legumes	0.32 *	−0.06	0.14	−0.02	0.30	0.10	0.37	−0.14	0.15	0.29 *	-		
12. fruits	0.76 *	−0.05	0.52 *	0.29 *	0.32 *	0.34 *	0.37 *	0.17	0.23	0.26	0.18	-	
13. vegetables	0.68 *	−0.14	0.22	0.10	0.47 *	0.06	0.33	0.07	0.40 *	0.38 *	0.37 *	0.55 *	-
Descriptive statistics of consumption frequency and related variables (median, Q1, Q3)													
Median	25.50	50.00	4.0	0.14	0.14	0.50	0.50	0.14	0.50	0.14	0.14	1.00	1.00
Q_1_	17.30	43.00	3.0	0.06	0.06	0.06	0.06	0.06	0.14	0.06	0.06	0.50	0.50
Q_3_	40.30	58.00	4.0	0.50	1.00	1.00	1.00	0.50	0.50	0.14	0.14	2.00	2.00

Spearman’s rank correlation coefficient, *—statistically significant, *p* < 0.05—a statistically significant value.

**Table 3:** Determinants of diet quality of masters athletics from France.

	1.	2.	3.	4.	5.	6.	7.	8.	9.	10.	11.	12.	13.
1. diet quality	-												
2. age	0.19	-											
3. number of meals daily	−0.39	0.11	-										
4. wholemeal bread	0.64 *	0.03	−0.13	-									
5. grains and ground	0.48	−0.23	0.24	0.69 *	-								
6. milk	0.23	−0.14	0.43	0.00	0.33	-							
7. fermented milk beverages	0.78 *	0.19	−0.64 *	0.55 *	0.22	−0.53	-						
8. curd	0.82 *	0.42	−0.15	0.79 *	0.56 *	−0.07	0.65 *	-					
9. white meat	0.72 *	0.06	−0.24	0.25	0.34	0.18	0.41	0.42	-				
10. fish	0.29	−0.13	−0.14	0.37	0.38	0.01	0.12	0.11	0.21	-			
11. legumes	0.66 *	0.24	−0.06	0.38	0.55 *	0.17	0.43	0.56 *	0.53 *	0.50	-		
12. fruits	0.57 *	0.01	−0.14	0.41	0.20	−0.31	0.44	0.31	0.25	0.31	0.47	-	
13. vegetables	0.52	0.22	−0.38	0.14	0.05	−0.20	0.47	0.51	0.34	−0.41	0.08	−0.10	-
Descriptive statistics of consumption frequency and related variables (median, Q1, Q3)													
Median	29.75	52.00	4.0	0.50	0.50	0.06	0.32	0.32	0.50	0.50	0.14	2.00	2.00
Q_1_	24.60	41.00	3.0	0.00	0.06	0.00	0.14	0.06	0.14	0.14	0.14	1.00	1.00
Q_3_	35.70	57.00	4.0	1.00	0.50	0.14	1.00	0.50	1.00	0.50	0.50	2.00	2.00

Spearman’s rank correlation coefficient, *—statistically significant, *p* < 0.05—a statistically significant value.

**Table 4:** Determinants of diet quality of masters athletics from Great Britain.

	1.	2.	3.	4.	5.	6.	7.	8.	9.	10.	11.	12.	13.
1. diet quality	-												
2. age	−0.31	-											
3. number of meals daily	0.05	0.15	-										
4. wholemeal bread	0.32	−0.44 *	−0.02	-									
5. grains and groats	0.08	−0.28	−0.14	0.30	-								
6. milk	0.33	−0.16	−0.59 *	0.08	−0.20	-							
7. fermented milk beverages	0.21	0.14	0.14	0.25	−0.39	0.06	-						
8. curd	−0.05	0.04	0.45 *	0.03	−0.24	−0.24	0.36	-					
9. white meat	−0.20	−0.04	0.03	−0.10	−0.47 *	0.20	−0.09	−0.03	-				
10. fish	0.35	0.14	0.42	−0.25	0.00	−0.29	−0.11	0.11	0.09	-			
11. legumes	0.35	−0.20	0.27	−0.24	−0.25	−0.03	−0.04	0.33	−0.11	0.10	-		
12. fruits	0.61 *	−0.12	−0.22	0.05	0.30	0.10	−0.03	−0.16	−0.27	0.10	0.15	-	
13. vegetables	0.52 *	−0.09	0.06	0.14	−0.04	0.13	−0.30	−0.30	−0.11	0.19	0.19	0.16	-
Descriptive statistics of consumption frequency and related variables (median, Q1, Q3)													
Median	31.00	50.00	3.0	0.50	0.14	0.06	0.50	0.06	0.50	0.14	0.14	1.00	2.00
Q_1_	26.60	44.00	3.0	0.00	0.06	0.06	0.06	0.00	0.14	0.14	0.06	1.00	1.00
Q_3_	36.30	57.00	4.0	1.00	0.50	1.00	1.00	0.50	0.50	0.50	0.50	2.00	2.00

Spearman’s rank correlation coefficient, *—statistically significant, *p* < 0.05—a statistically significant value.

**Table 5:** Regression analysis predicting diet quality in masters athletics from Poland, France, and Great Britain.

Variable	R^2^	β	F	*p* Value
Model 1: Poland	0.992		645.67	<0.001
fruits		0.27		<0.001
grains and coarse-ground groats		0.19		<0.001
curd		0.15		<0.001
vegetables		0.27		<0.001
milk		0.28		<0.001
wholemeal bread		0.25		<0.001
white meat		0.17		<0.001
fermented milk beverages		0.19		<0.001
Model 2: France	0.898		29.31	<0.001
curd		0.67		<0.001
white meat		0.30		0.018
fruits		0.28		0.027
Model 3: Great Britain	0.522		9.83	0.001
fruits		0.57		0.003
vegetables		0.35		0.047

Multiple step regression, *p* < 0.05—a statistically significant value.

## Data Availability

The data presented in this study are available on the request from the corresponding author.
